# Dynamical analysis of antibody aggregation in the CHO cell culture with Thermo Responsive Protein A (TRPA) column

**DOI:** 10.1186/1753-6561-7-S6-P69

**Published:** 2013-12-04

**Authors:** Masahiro Noda, Masayoshi Onitsuka, Miki Tatsuzawa, Ichiro Koguma, Takeshi Omasa

**Affiliations:** 1 Graduate School of Advanced Technology and Science, The University of Tokushima, Tokushima, 770-8506, Japan; 2 Institute of Technology and Science, The University of Tokushima, Tokushima, 770-8506, Japan; 3 New Products Development Department, Asahikasei Medical Co., LTD., Bioprocess Division, Fuji, 416-8501, Japan

## Background

Aggregation of therapeutic antibody is generally occurred in its manufacturing process, and should be suppressed and removed because its potential risk for unexpected immune response [1,2]. Protein A affinity chromatography is the first purification step in the monoclonal antibody manufacturing. Although the affinity purification is a powerful technique, high affinity between protein A and antibody requires acidic condition (below pH 3.0) to elute the captured antibody molecules. Exposure to acidic condition can induce the denaturation and aggregation of antibody molecules, demonstrating the necessity of novel strategy to reduce the antibody aggregation in the affinity purification process. Here we introduced a novel affinity purification strategy, thermo responsive protein A (TRPA) resin. TRPA is an engineered protein A ligand which adopts folded structure under 10°C and unfolds at moderate temperature, above 25°C. TRPA resin can control capture and elution of antibody by changing column temperature, making it possible to elute antibody molecules without low pH condition. In this study, we applied the TRPA column to the purification of Ex3 humanized IgG-like single-chained bispecific diabody-Fc (Ex3-scDb-Fc) [3]. The bispecific diabody is the promising candidate for next-generation therapeutic antibody, whereas it shows aggregation tendency. Furthermore, we observed the time-dependent formation of antibody aggregation in the culture process of the recombinant Chinese hamster ovary (CHO) cell line with TRPA column.

## Materials and methods

CHO Top-H cell line producing the Ex3-scDb-Fc [4] was cultivated in a 1L-glass bioreactor with working volume of 750mL serum-free medium. Viable cell densities and antibody concentrations in the medium was determined with Vi-Cell XR™ cell viability analyzer (Beckman Coulter) and by ELISA, respectively. The bispecific diabody was purified with conventional protein A (PA) column or thermo responsive protein A (TRPA) column, which were connected with AKTA prime plus (GE Healthcare). Elution of antibody was performed by acidic pH solution (pH2.7) for PA column and by raising column temperature to 45°C for TRPA column. Aggregate formation was analyzed with Superdex 200 10/30 GL column (GE Healthcare).

## Results and discussion

### Performance of TRPA column in the affinity purification of bispecific diabody-Fc

**Figure 1:**
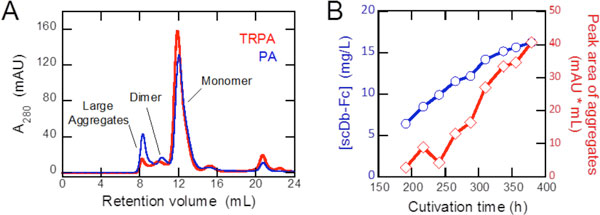
**(A) Size-exclusion chromatography showing the elution profiles of Ex3-scDb-Fc purified with TRPA (red) and PA (blue)**. **(B) **Time-dependent formation of aggregates in CHO cell culture.

We purified the Ex3-scDb-Fc from the culture supernatant of CHO Top-H cell line with PA and TRPA column. Compared to the conventional protein A column (PA), purification with TRPA column showed no precipitation of the aggregated scDb-Fc after the elution. Figure 1A is the size exclusion chromatography (SEC) profiles, showing that TRPA purification substantially reduced the formation of soluble large aggregates as compared to the PA purification including the exposure to acidic pH condition. Collectively, the above results demonstrate that TRPA column is highly effective in preventing the formation of precipitated and soluble aggregates in the affinity purification of the bispecific diabody-Fc.

### Dynamical aggregation analysis in the cell culture process

SEC profile of TRPA-purified Ex3-scDb-Fc would correctly reflect the status of antibody aggregation in CHO cell culture, because no further aggregation was induced in the affinity purification process with TRPA column as compared with that with conventional PA. Although secreted antibody is known to aggregate during cell culture process [1,2], the underlying mechanism is still poorly understood due to the lack of observation of the aggregation process. We applied the TRPA column to dynamical aggregation analysis of Ex3-scDb-Fc in CHO cell culture. Culture supernatants from exponential to stationary growth phase in a bioreactor operation were sampled, and the bispecific diabody was purified with TRPA column and analyzed by Size exclusion chromatography. The procedure makes it possible to observe the time-dependent formation of antibody aggregates in CHO cell culture. In Figure 1B, the peak areas of large aggregates were plotted as a function of cultivation time, showing that after 250 hours the amounts of aggregated Ex3-scDb-Fc were abruptly increased in time dependent manner. The results suggest a nucleation-dependent aggregation model for antibody aggregation, where the accumulation of aggregation nucleus is the rate limiting step and then the nucleus induces the formation of large aggregates in CHO cell culture. The bispecific diabody in this study has a tendency to aggregate during the CHO cell culture process, demonstrating the necessity of the novel cell culture strategy to suppress the aggregates formation.

## Conclusions

We propose the Thermo Responsive Protein A (TRPA) column as a novel strategy to reduce the antibody aggregation in an affinity purification process and to analysis the aggregation during the cell culture process.
